# Early dialysis initiation does not improve clinical outcomes in elderly end-stage renal disease patients: A multicenter prospective cohort study

**DOI:** 10.1371/journal.pone.0175830

**Published:** 2017-04-17

**Authors:** Jae Yoon Park, Kyung Don Yoo, Yong Chul Kim, Dong Ki Kim, Kwon Wook Joo, Shin-Wook Kang, Chul Woo Yang, Nam-Ho Kim, Yong-Lim Kim, Chun-Soo Lim, Yon Su Kim, Jung Pyo Lee

**Affiliations:** 1 Department of Internal Medicine, Dongguk University Ilsan Hospital, Gyeonggi-do, Korea; 2 Department of Internal Medicine, Dongguk University Medical Center, Gyeongsangbuk-do, Korea; 3 Department of Internal Medicine, Seoul National University College of Medicine, Seoul, Korea; 4 Department of Internal Medicine, Yonsei University College of Medicine, Seoul, Korea; 5 Department of Internal Medicine, The Catholic University of Korea College of Medicine, Seoul, Korea; 6 Department of Internal Medicine, Chonnam National University Medical School, Gwangju, Korea; 7 Department of Internal Medicine, Kyungpook National University School of Medicine, Daegu, Korea; 8 Department of Internal Medicine, Seoul National University Boramae Medical Center, Seoul, Korea; The University of Tokyo, JAPAN

## Abstract

**Background:**

The optimal timing for initiating dialysis in end-stage renal disease (ESRD) is controversial, especially in the elderly.

**Methods:**

665 patients ≥65 years old who began dialysis from August 2008 to February 2015 were prospectively enrolled in the Clinical Research Center for End-Stage Renal Disease cohort study. Participants were divided into 2 groups based on the median estimated glomerular filtration rate at the initiation of dialysis. Propensity score matching (PSM) was used to compare the overall survival rate, cardiovascular events, Kidney Disease Quality of Life Short Form 36 (KDQOL-36) results, Karnofsky performance scale values, Beck’s depression inventory values, and subjective global assessments.

**Results:**

The mean patient age was 72.0 years, and 61.7% of the patients were male. Overall, the cumulative survival rates were lower in the early initiation group, although the difference was not significant after PSM. Additionally, the survival rates of the 2 groups did not differ after adjusting for age, sex, Charlson comorbidity index and hemoglobin, serum albumin, serum calcium and phosphorus levels. Although the early initiation group showed a lower physical component summary score on the KDQOL-36 3 months after dialysis, the difference in scores was not significant 12 months after dialysis. Furthermore, the difference was not significant after PSM. The Karnofsky performance scale, Beck’s depression inventory, and subjective global assessments were not significantly different 3 and 12 months after dialysis initiation.

**Conclusions:**

The timing of dialysis initiation is not associated with clinical outcomes in elderly patients with ESRD.

## Introduction

Elderly individuals represent the fastest-growing population of incident dialysis patients worldwide [1–3]. However, the ideal timing of dialysis initiation in this group is not known, and patients in this age group are more likely to present multiple comorbidities [4]. Dialysis initiation may improve the nutritional status and survival of patients through increased uremic solute clearance. Early initiation strategies have been supported since 1995 [5], and conventional wisdom indicates that delaying dialysis is potentially dangerous. Although specific criteria for dialysis initiation are not available for elderly patients, until the late 2000s, treatment was initiated in the earlier stages of kidney dysfunction, which is similar to the procedure for other age groups [6]. However, after the first randomized controlled trial (RCT) regarding the timing of dialysis initiation and clinical outcomes, the early initiation of dialysis was challenged because expert recommendations no longer supported this early initiation strategy [7]. In addition, the importance of a palliative approach is emphasized in the elderly end-stage renal disease (ESRD) population because of the burden of treatment and its negative effect on quality of life (QOL). Systematic assessments in the elderly, including cognitive, functional and psychosocial issues, should also be considered in the context of dialysis initiation. Nonetheless, prospective studies on the start of dialytic therapy in elderly patients with ESRD are limited, especially in Asian populations.

Therefore, we examined the effect of dialysis initiation timing on clinical outcomes, such as mortality, morbidity, and QOL benefits, in elderly patients in the Clinical Research Center for End-Stage Renal Disease (CRC for ESRD) cohort.

## Materials and methods

### Study participants

**Fig 1:**
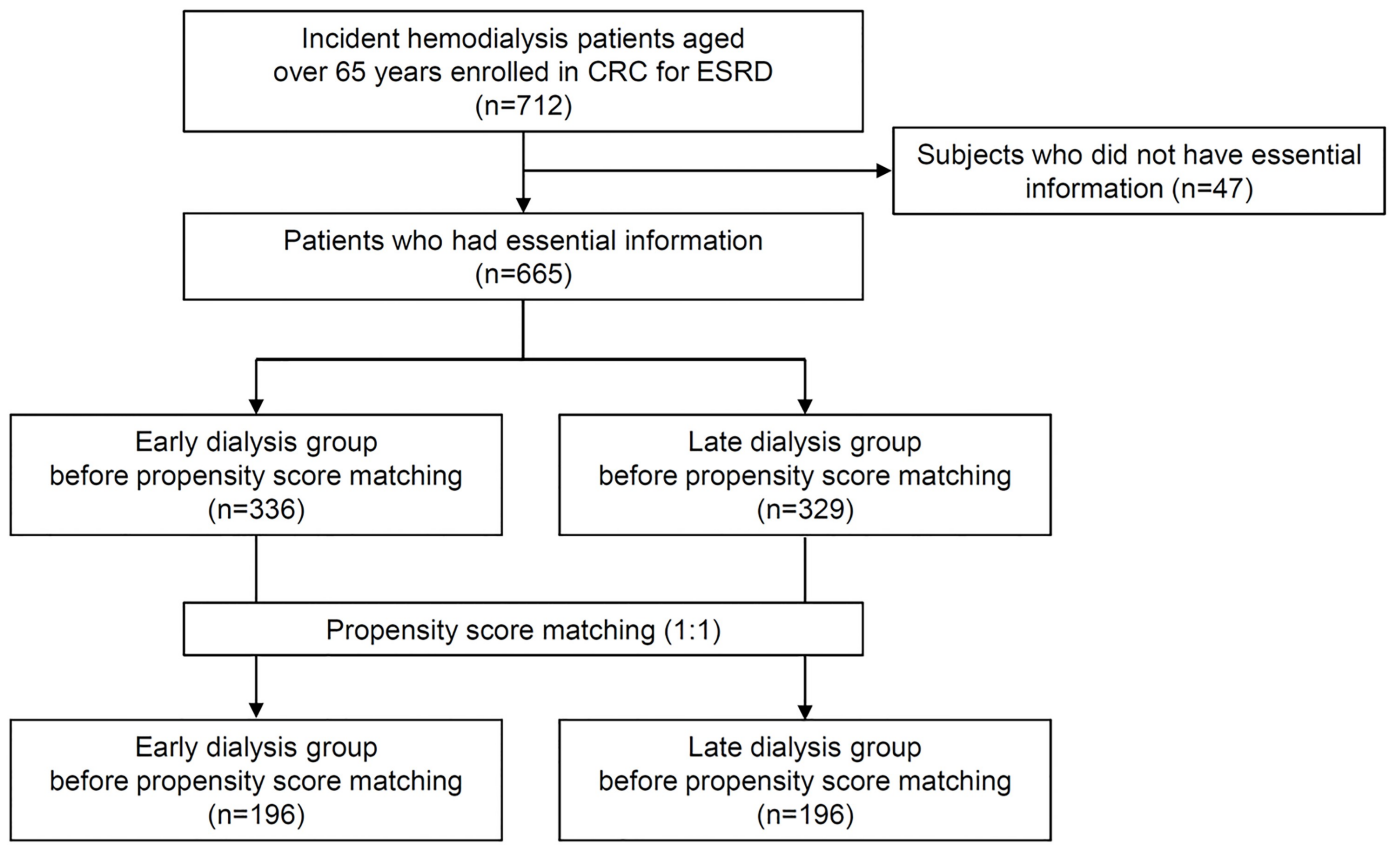
Flow chart of the study.

The CRC for ESRD cohort is a nationwide, multi-center, prospective cohort of ESRD patients undergoing dialysis in South Korea [8, 9]. The CRC for ESRD cohort began registering ESRD patients for dialysis in July 2008, and 31 hospitals in South Korea are currently participating. Patients aged 65 years or older who started dialysis for ESRD between July 2008 and February 2015 were eligible for the study (Fig 1).

### Data collection

Data were extracted from the CRC for ESRD database (http://webdb.crc-esrd.co.kr) for the outcome analysis. The baseline information included age, sex, height, weight, primary renal disease, Charlson co-morbidity index (CCI), Karnofsky performance status (KPS), subjective global assessment (SGA), and laboratory data. Comorbidities, laboratory data, 24-hour urine volume and dialysis information were followed at 3 and 6 months after the start of dialysis and at 6-month intervals thereafter. Laboratory data and 24-hour urine volume were analyzed using time-averaged values. The dialysis modality was defined as the modality 3 months after the first dialysis treatment or the modality at dialysis initiation if death occurred before 3 months. The estimated glomerular filtration rate (eGFR) was calculated using Chronic Kidney Disease-Epidemiology Collaboration equations immediately prior to renal replacement therapy (RRT) [10]. Ambulation state was recorded in four categories: normal, walks with assistance (e.g., a person, cane or walker), requires a wheelchair and bed-ridden.

KPS and SGA data were followed at 12-month intervals. The KPS data were used to assess subject performance status and were defined as follows: KPS score ≥80: able to conduct normal activity and work, with no special care required; 70–50: unable to work and able to live at home and care for most personal needs, with varying amount of assistance required; KPS ≤40: unable to care for self and requires the equivalent of institutional or hospital care, and the disease may be progressing rapidly. For the nutritional status evaluation, the SGA scores were divided into 3 categories (1: well-nourished [SGA score, 6–7]; 2: mildly-to-moderately malnourished [3–5]; and 3: severely malnourished [1–2]). The number of subjects classified as category 3 was small; therefore, we classified the 3 SGA categories into 2 groups (category 1 versus categories 2 and 3).

### Clinical outcomes

The primary outcome was all-cause mortality after the start of dialysis. The secondary outcomes were cardiovascular events and the 1-year changes in the Kidney Disease Quality of Life-36 (KDQOL-36) survey, KPS values, Beck’s depression inventory (BDI) values, and SGA scores.

Cardiovascular events included clinical events requiring admission for ischemic heart disease, congestive heart failure, arrhythmia, or cerebrovascular disease.

### Survey instruments

The KDQOL-36 survey was used to evaluate the health-related QOL of the ESRD patients [11]. We utilized the Korean version [12], which includes 12 generic chronic disease items (short form [SF]-12) and 24 additional kidney disease-targeted items (symptom/problem list, 12 items; effects of kidney disease, 8 items; and burden of disease, 4 items). The item scores were aggregated without weighting and transformed linearly to a 0–100 range, with higher scores indicating better states.

The Korean version of the BDI was used to evaluate depression [13]. The BDI consists of 21 self-reported items rated on a scale from 0–3, resulting in a possible score range of 0–63, with higher scores indicating more severe depression.

### Statistical analysis

Continuous variables were expressed as the mean and standard deviation, and categorical variables were presented as frequencies with percentages. Continuous variables were compared using a t-test, and categorical variables were compared using the Chi-square test or Fisher's exact test. Survival was compared using the Kaplan-Meier curve and log-rank test. Propensity scores were estimated using a multiple logistic regression analysis adjusted for patient age, sex, primary renal disease, CCI and hemoglobin, albumin, calcium, and phosphorus levels. After determining the propensity scores, we matched the patients in the early and late dialysis groups with similar propensity scores at a 1:1 ratio using the nearest neighbor method without replacements and a 0.2 caliper width. Propensity score matching (PSM) was used to increase the precision of the estimated effect without increasing bias because certain variables were potentially associated with survival [14]. The characteristics of both the early and late dialysis groups were compared before and after PSM. The Kaplan-Meier survival curves and life tables were estimated for the early and late dialysis groups after PSM.

All of the statistical tests were evaluated using a two-tailed 95% confidence interval (CI), and *P*<0.05 was considered statistically significant. All of the descriptive and survival analyses were performed using SPSS for Windows, version 21.0 (IBM, Armonk, NY, USA). R software (version 2.14.2) was used for PSM.

### Ethical aspects

The study protocol complied with the Declaration of Helsinki and received full approval from the institutional review board (IRB) at Seoul National University Hospital (H-1405-060-579). The study protocol of the CRC for ESRD was approved by the IRB at each participating center, and all of the patients provided their written informed consent.

## Results

### Participant characteristics

The baseline characteristics of the 665 patients are listed in Table 1. At the initiation of dialysis, the mean patient age was 72.0 ± 5.4 years, and the patients were 61.7% male. The patients were divided into 2 groups based on the median eGFR immediately prior to the initiation of dialysis. The median eGFR before initiating dialysis was 8.8 mL/min/1.73 m^2^. Prior to PSM, 336 patients were in the early dialysis group, and 329 patients were in the late dialysis group. The numbers of patients underwent hemodialysis (HD) and peritoneal dialysis (PD) in the early versus late dialysis groups were 277 (82.4%) and 59 (17.6%) versus 284 (86.3%) and 45 (13.7%), respectively. There was no statistical difference in the proportion of patients underwent each dialysis modality.

**Table 1:** Baseline characteristics before and after propensity score matching at the start of dialysis in the 2 groups based on the timing of dialysis initiation stratified by the median eGFR before dialysis.

Variables	Before propensity score matching				After propensity score matching			
	Early dialysis (N = 336)	Late dialysis (N = 329)	*P* value	SD	Early dialysis (N = 196)	Late dialysis (N = 196)	*P* Value	SD
Age (years)	72.8 ± 5.7	71.2 ± 4.9	<0.001	0.293	71.7 ± 5.3	72.1 ± 5.0	0.511	-0.060
Sex, male (n [%])	180 (53.6%)	230 (69.9%)	<0.001	-0.314	118 (60.2%)	124 (63.3%)	0.603	-0.061
Body mass index (kg/m^2^)	23.0 ± 3.5	22.8 ± 3.4	0.454		23.3 ± 3.8	23.0 ± 3.7	0.439	
Primary renal disease			0.004	-0.282			0.740	-0.039
Diabetic nephropathy	230 (69.5%)	184 (56.3%)			126 (64.3%)	123 (62.8%)		
Hypertensive nephropathy	57 (17.2%)	72 (22.0%)			40 (20.4%)	43 (21.9%)		
Glomerulonephritis	18 (5.4%)	29 (8.9%)			14 (7.1%)	10 (5.1%)		
Others	26 (7.9%)	42 (12.8%)			16 (8.2%)	20 (10.2%)		
Charlson comorbidity index	7.3 ± 2.1	6.4 ± 1.7	<0.001	0.436	7.0 ± 2.0	6.7 ± 1.6	0.228	0.105
Ambulation			0.241				0.093	
Normal	234 (69.9%)	240 (73.4%)			138 (70.4%)	141 (72.3%)		
Walks with assistance	62 (18.5%)	46 (14.1%)			37 (18.9%)	25 (12.8)		
Requires wheelchair	23 (6.9%)	30 (9.2%)			12 (6.1%)	23 (11.8%)		
Bed-ridden	16 (4.8%)	11 (3.4%)			9 (4.6%)	6 (3.1%)		
Systolic blood pressure (mmHg)	142.2 ± 21.5	143.1 ± 24.1	0.634		141.7 ± 20.7	143.7 ± 23.7	0.384	
Diastolic blood pressure (mmHg)	73.8 ± 12.4	73.3 ± 12.9	0.611		74.1 ± 12.1	73.6 ± 12.8	0.671	
Biochemical data								
Hemoglobin (g/dL)	9.2 ± 1.5	8.8 ± 1.4	<0.001	0.279	9.0 ± 1.4	9.1 ± 1.3	0.624	-0.044
Albumin (g/dL)	3.2 ± 0.6	3.4 ± 0.5	0.005	-0.215	3.3 ± 0.6	3.3 ± 0.5	0.815	0.022
Blood urea nitrogen (mg/dL)	59.9 ± 31.2	96.5 ± 36.6	<0.001		64.9 ± 29.5	86.4 ± 31.9	<0.001	
Creatinine (mg/dL)	5.0 ± 1.2	9.3 ± 1.6	<0.001		5.2 ± 1.1	8.6 ± 3.3	<0.001	
eGFR (mL/min/1.73m^2^)	13.5 ± 6.5	6.5 ± 1.6	<0.001		12.5 ± 4.8	7.0 ± 1.3	<0.001	
Potassium (mEq/L)	4.4 ± 1.0	4.7 ± 0.9	0.002		4.6 ± 1.0	4.6 ± 0.8	0.962	
Calcium (mg/dL)	8.1 ± 0.8	7.8 ± 0.9	<0.001	0.330	8.0 ± 0.8	8.0 ± 0.8	0.486	0.073
Phosphorus (mg/dL)	4.5 ± 3.4	5.7 ± 1.8	<0.001	-1.036	4.7 ± 1.3	4.9 ± 1.2	0.249	-0.113
Cholesterol (mg/dL)	148.4 ± 40.8	149.7 ± 45.9	0.716		145.7 ± 36.0	150.4 ± 46.9	0.286	
Transferrin saturation (%)	29.3 ± 21.9	35.9 ± 50.1	0.040		28.6 ± 18.3	31.0 ± 30.0	0.362	
Intact parathyroid hormone (pg/mL)	181.1 ± 144.4	248.9 ± 225.8	<0.001		195.0 ± 150.1	232.6 ± 168.5	0.036	
Cardiothoracic ratio over 50% (n[%])	107 (35.9%)	110 (36.8%)	0.865		70 (39.1%)	65 (36.9%)	0.743	

Abbreviations: eGFR, estimated glomerular filtration rate; SD, standardized difference

The mean body mass index, systolic blood pressure, and diastolic blood pressure did not differ; however, the primary ESRD diseases differed between the 2 groups (P = 0.004). Diabetic nephropathy was a more common primary disease in the early dialysis group than in the late dialysis group (62.9% versus 56.3%). The CCI, hemoglobin concentration, eGFR and calcium serum level were higher, and the serum albumin, blood urea nitrogen, creatinine, potassium, phosphorus, intact parathyroid hormone levels and transferrin saturation were lower in the early dialysis group. Differences in the ambulation state, serum brain natriuretic peptide (BNP) and cholesterol levels and the number of patients with cardiothoracic ratio over 50% on simple chest X-ray were not observed between the 2 groups.

All of the patients in both groups were matched by the propensity score for dialysis initiation timing using the following covariates: age, sex, primary renal disease, CCI and hemoglobin, albumin, calcium, and phosphorus levels. After PSM, 392 patients (196 in each group) remained. The distribution of the propensity scores before and after matching is shown in the supplementary data (S1 Fig). Of the patients subjected to PSM, almost all of the baseline parameters, including age, sex, body mass index, primary renal disease, CCI, ambulation state, systolic blood pressure, diastolic blood pressure, biochemical data (except blood urea nitrogen, creatinine, eGFR and intact parathyroid hormone) and cardiothoracic ratio over 50% on simple chest X-ray, were similar between the early and late dialysis groups. The propensity scores of the matched patients were not different between the groups.

In hemodialysis patients, there were no differences in dialysis frequency per week (2.8 ± 0.5 versus 2.9 ± 0.5), time per session (3.9 ± 0.6 versus 3.9 ± 0.5), urea reduction ratio (68.8 ± 8.7 versus 69.6 ± 8.8) or Kt/V (1.4 versus 1.5) 3 months after dialysis initiation (S1 Table). In peritoneal dialysis patients, there was no difference in weekly Kt/V (3.2 ± 2.7 versus 3.1 ± 3.0) 3 months after dialysis initiation.

### Patient survival and cardiovascular event-free survival analyses stratified by dialysis initiation timing

**Fig 2:**
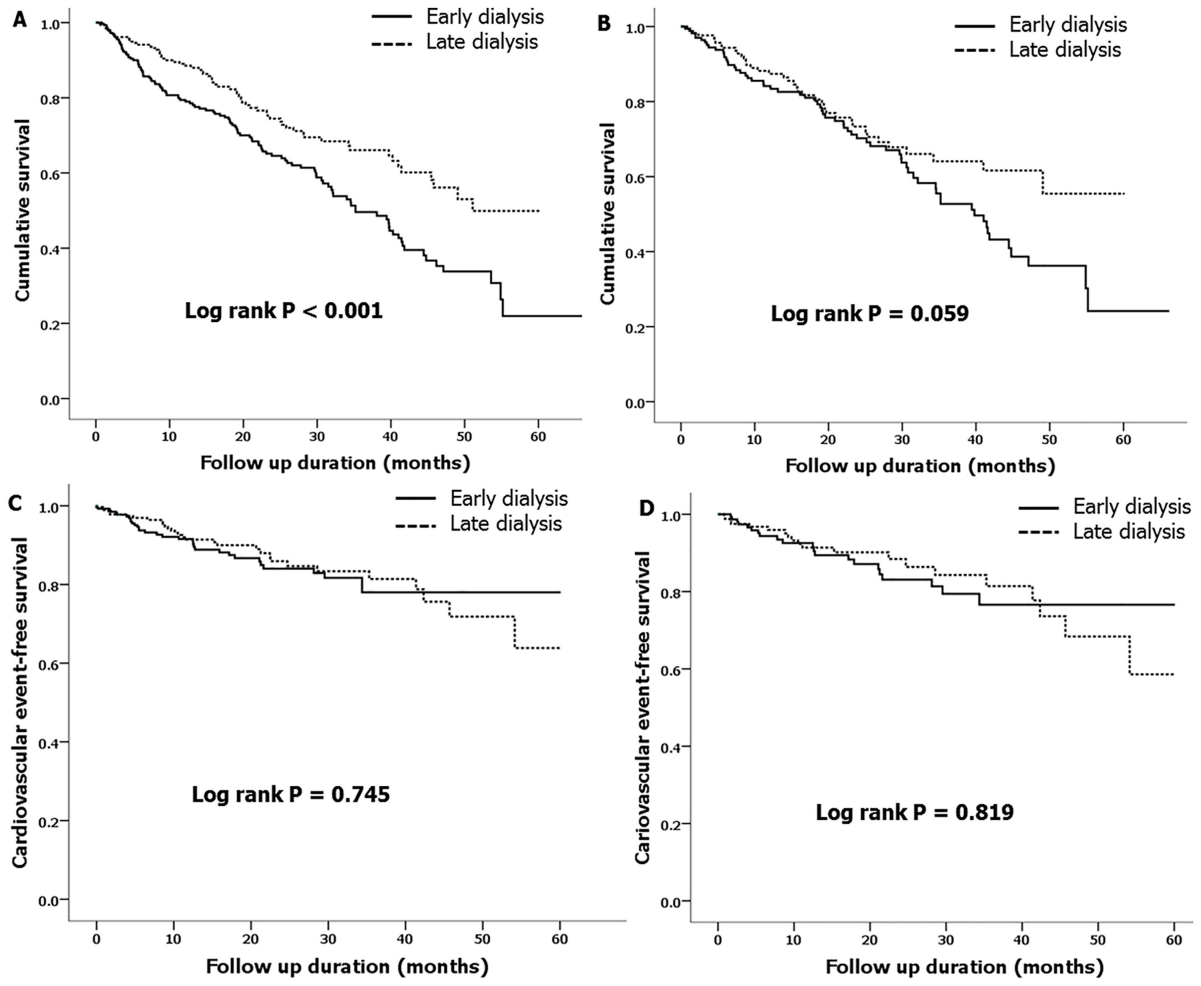
Cumulative patient survival (A and B) and cardiovascular event-free survival (C and D) obtained using the Kaplan-Meier method in the cohort differentiated by the median estimated glomerular filtration rate before (A and C) and after (B and D) propensity score matching.

A total of 188 mortalities (28.3%) were observed during the median follow-up period of 12.8 months. Fig 2A and 2B show the survival curves obtained using the Kaplan-Meier method, which were differentiated by the median eGFR. The early dialysis group showed a significantly lower cumulative survival rate than the late dialysis group; however, significances were not observed after PSM. Univariate and multivariate Cox regression analyses were also performed (Table 2). Before PSM, the unadjusted model revealed that the early dialysis group had an increased hazard ratio (HR) for mortality (Model 1: HR 1.70, 95% CI 1.26–2.89, P<0.001). This association was significant after adjusting for age, sex, and CCI (Model 2: HR 1.37, 95% CI 1.00–1.27, P = 0.049). However, this association was not significant after adjusting for hemoglobin, albumin, calcium, and phosphorus levels (Model 3: HR 1.30, 95% CI 0.89–1.89, P = 0.176). Additionally, the association was not significant after PSM (Model 4: HR 1.46, 95% CI 0.98–2.20, P = 0.064).

**Table 2:** Multivariate risk factor analysis of the mortality of elderly patients with end-stage renal disease in the early dialysis group compared to the late dialysis group using Cox regression models and a propensity score matched model.

	Hazard ratio	95% confidence interval	*P* value
Model 1^a^	1.70	1.26–2.89	<0.001
Model 2^b^	1.37	1.00–1.27	0.049
Model 3^c^	1.30	0.89–1.89	0.176
Model 4^d^	1.46	0.98–2.20	0.064

^a^Unadjusted

^b^Adjusted for age, sex, and Charlson comorbidity index

^c^Adjusted for age, sex, Charlson comorbidity index, hemoglobin, albumin, calcium, and phosphorus

^d^Propensity score matched; covariates for matching: age, sex, Charlson comorbidity index, hemoglobin, albumin, calcium, and phosphorus

Fig 2C and 2D show the cardiovascular event-free survival curves. Differences in the cumulative cardiovascular event-free survival rates were not observed before and after PSM.

In the hemodialysis subgroup of 561 patients, the early initiation group showed lower survival rates before PSM, but no significant difference after PSM. Additionally, the cumulative cardiovascular event-free survival rates did not significantly differ between the 2 groups before and after PSM. In the peritoneal dialysis subgroup of 104 patients, differences in the cumulative patient or cardiovascular event-free survival rates were not observed before or after PSM.

In the older subgroup of 408 patients over 70 years of age, the early dialysis group showed significantly lower cumulative survival rates than the late dialysis group (S2A Fig). This difference remained significant after PSM (S2B Fig). The cumulative cardiovascular event-free survival rates did not differ between the 2 groups before (S2C Fig) or after PSM (S2D Fig). Univariate and multivariate Cox regression analyses were also conducted (S2 Table). In the unadjusted model, the early dialysis group had an increased HR for mortality (Model 1: HR 1.82, 95% CI 1.24–2.65). Although this association was marginal after adjusting for age, sex and CCI (Model 2: HR 1.48, 95% CI 1.00–2.20) and after adjusting for hemoglobin, albumin, calcium and phosphorus levels (Model 3: HR 1.49, 95% CI 1.00–2.22), it was significant after PSM (Model 4: HR 1.91, 95% CI 1.16–3.16).

### Analyses of quality of life, nutritional status and physical and mental performances

Regarding the KDQOL-36, SGA, KPS, and BDI scores determined according to dialysis initiation timing, differences were not observed between the groups both at 3 months and 1 year from the initiation of dialysis except for in the physical component summary of the 5 KDQOL domains (Table 3), which was lower in the early dialysis group at 3 months after dialysis initiation (34.0 ± 8.6 versus 38.2 ± 9.1, P = 0.010). However, the difference was not significant at 1 year after dialysis initiation. Furthermore, any differences in the KDQOL-36, SGA, KPS, and BDI scores were not observed at 3 months and 1 year after dialysis initiation after PSM.

**Table 3:** Analyses of quality of life, nutritional status, and physical and mental performance using the KDQOL-36 survey, subjective global assessments, Karnofsky performance scale values, and Beck’s depression inventory scores before and after propensity score matching at 3 months versus 1 year after dialysis initiation in the 2 groups based on the timing of dialysis initiation stratified by the median eGFR before dialysis.

Variables	Before propensity score matching			After propensity score matching		
	Early dialysis (N = 336)	Late dialysis (N = 329)	*P* value	Early dialysis (N = 196)	Late dialysis (N = 196)	*P* Value
At 3 months after dialysis						
KDQOL-36						
Physical component summary	34.0 ± 8.6	38.2 ± 9.1	0.010	34.9 ± 9.4	36.7 ± 9.6	0.379
Mental component summary	41.9 ± 9.6	40.0 ± 10.0	0.291	43.2 ± 8.9	39.8 ± 10.1	0.114
Symptom/problem list	76.3 ± 17.4	76.9 ± 17.0	0.846	77.5 ± 17.8	73.8 ± 17.9	0.358
Effect of kidney disease	66.3 ± 18.3	66.6 ± 19.7	0.935	67.2 ± 19.8	62.9 ± 20.8	0.345
Burden of kidney disease	25.4 ± 20.0	28.4 ± 21.6	0.423	24.1 ± 20.6	27.8 ± 20.2	0.426
Subjective global assessment			0.339			1.000
Well-nourished (n [%])	146 (75.3%)	162 (79.4%)		87 (75.7%)	93 (76.2%)	
Mildly-to-moderately malnourished (n [%])	48 (24.7%)	42 (20.6%)		28 (23.8%)	29 (23.8%)	
Severely malnourished (n [%])	-	-		-	-	
Karnofsky performance scale	71.8 ± 15.7	73.9 ± 15.5	0.265	73.3 ± 15.6	71.7 ± 15.5	0.510
Beck’s depression inventory	17.9 ± 10.6	16.6 ± 11.5	0.394	17.1 ± 9.7	17.1 ± 11.6	0.988
At 1 year after dialysis						
KDQOL-36						
Physical component summary	36.3 ± 10.4	37.6 ± 9.7	0.576	36.5 ± 11.4	38.5 ± 10.0	0.521
Mental component summary	41.1 ± 10.3	42.7 ± 7.8	0.459	40.7 ± 10.2	44.1 ± 6.9	0.211
Symptom/problem list	81.5 ± 13.9	78.0 ± 15.0	0.328	80.6 ± 14.7	79.2 ± 15.3	0.760
Effect of kidney disease	66.4 ± 20.4	70.0 ± 20.1	0.479	62.8 ± 20.5	73.9 ± 18.7	0.062
Burden of kidney disease	32.3 ± 29.2	36.2 ± 25.4	0.548	32.8 ± 32.4	35.6 ± 25.9	0.749
Subjective global assessment			0.282			0.267
Well-nourished (n [%])	94 (81.0%)	101 (87.1%)		54 (77.1%)	55 (85.9%)	
Mildly-to-moderately malnourished (n [%])	22 (19.0%)	15 (12.9%)		16 (22.9%)	9 (14.1%)	
Severely malnourished (n [%])	-	-		-	-	
Karnofsky performance scale	73.1 ± 16.2	73.9 ± 14.4	0.751	73.4 ± 15.8	74.8 ± 14.5	0.675
Beck’s depression inventory	18.1 ± 11.0	16.9 ± 9.9	0.523	17.7 ± 10.7	15.0 ± 8.0	0.200

Abbreviations: eGFR, estimated glomerular filtration rate; KDQOL-36, kidney disease quality of life short form 36

### Changes in laboratory findings and 24-hour urine output after 1 year of dialysis initiation

At 1 year after dialysis initiation, the blood chemistry and 24-hour urine volume were compared between the early and late dialysis groups and at 3 months after the start of dialysis (Table 4). Prior to PSM, the serum albumin level at 3 months after the start of dialysis was lower (3.5 ± 0.6 versus 3.6 ± 0.5, P = 0.049), and the high-sensitivity C-reactive protein (hs-CRP) level after 1 year was higher (4.3 ± 11.3 versus 1.8 ± 3.6, P = 0.024) in the early dialysis group. Nonetheless, the differences were not significant after PSM. Other laboratory parameters, such as the hemoglobin, uric acid, cholesterol, calcium, phosphorus, β2-microglobulin contents and transferrin saturation, were not different between the early and late dialysis groups before and after PSM. Moreover, differences were not observed in the 24-hour urine volume before and after PSM.

**Table 4:** Changes in the laboratory findings and 24-hour urine output before and after propensity score matching at 3 months versus 1 year after dialysis initiation in the 2 groups based on the timing of dialysis initiation stratified by the median eGFR before dialysis.

Variables	Before propensity score matching			After propensity score matching		
	Early dialysis (N = 336)	Late dialysis (N = 329)	*P* value	Early dialysis (N = 196)	Late dialysis (N = 196)	*P* Value
At 3 months after dialysis						
Hemoglobin (g/dL)	11.0 ± 1.5	10.8 ± 1.9	0.397	10.9 ± 1.5	10.9 ± 2.2	0.911
Albumin (g/dL)	3.5 ± 0.6	3.6 ± 0.5	0.049	3.6 ± 0.5	3.6 ± 0.5	0.993
Uric acid (mg/dL)	6.4 ± 1.8	6.7 ± 1.8	0.060	6.6 ± 1.8	6.6 ± 1.8	0.809
Cholesterol (mg/dL)	162.3 ± 46.1	158.0 ± 42.4	0.305	164.0 ± 42.7	158.4 ± 43.8	0.286
Calcium (mg/dL)	8.5 ± 0.8	8.5 ± 0.8	0.582	8.6 ± 0.8	8.5 ± 0.8	0.356
Phosphorus (mg/dL)	4.0 ± 1.2	4.2 ± 1.3	0.065	4.3 ± 1.2	4.1 ± 1.2	0.159
β2-microglobulin (μg/mL)	27.0 ± 31.1	34.4 ± 44.1	0.117	28.3 ± 37.5	32.2 ± 39.2	0.523
Transferrin saturation (%)	30.9 ± 15.7	31.2 ± 14.4	0.809	30.3 ± 14.8	32.2 ± 15.6	0.354
hsCRP (mg/dL)	5.9 ± 20.0	3.6 ± 12.8	0.178	5.3 ± 19.1	4.7 ± 16.0	0.814
24-hours urine volume (mL)	688.5 ± 807.7	624.0 ± 533.9	0.381	764.9 ± 911.8	636.8 ± 559.9	0.220
At 1 year after dialysis						
Hemoglobin (g/dL)	10.8 ± 1.3	10.9 ± 1.2	0.726	10.8 ± 1.3	11.0 ± 1.2	0.209
Albumin (g/dL)	3.7 ± 0.5	3.8 ± 0.6	0.168	3.7 ± 0.5	3.7 ± 0.6	0.923
Uric acid (mg/dL)	6.3 ± 2.1	6.4 ± 1.7	0.833	6.2 ± 1.8	6.1 ± 1.8	0.651
Cholesterol (mg/dL)	155.8 ± 46.9	159.7 ± 38.1	0.431	153.7 ± 40.4	162.3 ± 37.2	0.149
Calcium (mg/dL)	8.6 ± 0.8	8.7 ± 0.9	0.751	8.7 ± 0.8	8.8 ± 0.9	0.512
Phosphorus (mg/dL)	4.1 ± 1.3	4.2 ± 1.0	0.290	4.3 ± 1.3	4.1 ± 1.0	0.296
β2-microglobulin (μg/mL)	28.9 ± 25.9	34.7 ± 50.3	0.348	28.6 ± 28.2	31.6 ± 56.7	0.738
Transferrin saturation (%)	32.1 ± 13.9	33.2 ± 14.4	0.532	30.6 ± 12.1	34.8 ± 15.0	0.059
hsCRP (mg/dL)	4.3 ± 11.3	1.8 ± 3.6	0.024	4.3 ± 12.8	2.1 ± 4.2	0.187
24-hours urine volume (mL)	562.9 ± 607.2	561.1 ± 737.5	0.986	562.9 ± 515.5	620.2 ± 852.9	0.665

Abbreviations: eGFR, estimated glomerular filtration rate; hsCRP, high-sensitivity C-reactive protein

## Discussion

In the present study, we compared the outcomes of dialysis according to the timing of initiation stratified by the median eGFR immediately before starting dialysis in the elderly using data obtained for 665 patients aged ≥65 years from a multicenter prospective cohort. The results indicate that survival benefits did not occur for the early dialysis initiation in the elderly. In addition, advantages of early dialysis initiation were not observed for the cardiovascular event-free survival, QOL and residual renal function preservation. The data show that dialysis can be delayed until the eGFR value falls below 8.8 mL/min/1.73 m^2^ if the patients have tolerable clinical conditions for dialysis initiation.

Until the late 2000s, early dialysis initiation was a worldwide trend because of concerns related to the delay of dialysis, which may cause prolonged uremia and lead to fatal uremic complications, such as intractable hyperkalemia, pulmonary edema, pericarditis, and encephalopathy. This concern was supported by several studies that showed that decreased eGFR values at the time of dialysis initiation were associated with poor patient survival and nutritional status [5, 15, 16]. Consequently, clinical guidelines have recommended dialysis initiation at relatively high eGFR values (over 10 mL/min/1.73 m^2^) without supporting evidence, such as RCT values [17–20]. Instead, most studies have applied a retrospective design and used insufficient baseline data, such as demographic and comorbid conditions [21, 22]. Additionally, lead-time bias should be considered when interpreting the data from these studies. Improvements to the survival rate after early dialysis initiation could be related to the earlier timing of dialysis initiation and may not represent actual survival gains caused by the early initiation of dialysis.

The only RCT detailing the effects of dialysis timing initiation on survival was reported in 2010 [7], and in this study, a total of 828 patients underwent randomization and started dialysis when the eGFR value was 10.0 to 14.0 mL per minute (early-start group) or 5.0 to 7.0 mL per minute (late-start group). After a median follow-up period of 3.59 years, differences were not observed between the groups with regard to survival and adverse event frequency (e.g., cardiovascular events, infection, or complication of dialysis). Therefore, the results of the 2010 study are consistent with the findings of our study. Moreover, advantages in survival and cardiovascular event-free survival were not observed in our study with regard to the early initiation of dialysis. Consistent with the first RCT conducted on dialysis initiation timing and survival, recent observational studies have shown that early initiation does not provide a survival benefit [21], and it may even promote harmful outcomes [23–29]. Our study focused on elderly patients because few studies have provided evidence to support specific strategies in this population with regard to how the initiation timing of dialysis affects clinical outcomes.

Recently, two studies that investigated the association between the timing of dialysis initiation and survival in the elderly were reported [30, 31]. In 2014, a study using data from the US Renal Data System (USRDS) (n = 84,654) indicated that early dialysis initiation (eGFR ≥10 mL/min/1.73 m^2^) was associated with greater mortality and hospitalization than late initiation (eGFR<10 mL/min/1.73 m^2^) [30]. The study presented here is characterized by the results from national registry data. Although the national registry provides information on almost every new ESRD patient, this is also a disadvantage. Because diagnoses were obtained using computerized codes such as ICD-9 in this study, the outcome occurrence accuracy may be problematic except for mortality data, which were obtained from the National Death Index and available for 99.3% of deaths. Our research is a prospective multicenter cohort study, and all of the participating institutions could recruit patients and perform follow-up investigations on the clinical parameters (e.g., comorbidity, hospitalization, biochemical, quality of life and nutritional status data, which were collected using the KDQOL-36, KPS, BDI, and SGA) in a consistent manner because the protocol was shared. Although our study is not a RCT, the clinical impact results may be similar to those obtained using large size registry data because our study encompasses a variety of clinical outcomes, including QOL, physical and mental performances, nutritional status, and survival and cardiovascular events. In 2015, another study of elderly ESRD patients reported that early referral to nephrologists (from the time of diagnosis of ESRD to the time of first encounter with the nephrologist ≥3 months) reduces the risk of long-term mortality by up to 24% relative to that of late referral (<3 months) [31]; however, this study corresponded to evidence regarding pre-dialysis nephrology care rather than dialysis initiation timing. In this study, the eGFR difference between both groups was relatively small (approximately 1.2 mL/min/1.73 m^2^), and the baseline biochemical parameters were worse in the late referral group. Our study focused on dialysis initiation timing and used PSM and the multivariate Cox regression model to overcome differences in the baseline characteristics, which may ultimately influence the outcomes of both the early and late dialysis groups.

The decision to start dialysis in elderly ESRD patients is a difficult and personal one. Many nephrologists are uncomfortable discussing dialysis as a life-prolonging rather than a life-saving modality in elderly ESRD patients, especially with multiple comorbidities and the following high mortality rates. Because of the structural and functional changes in the kidneys, multiple comorbidities, and resulting medications, the elderly population may be less able to adapt to dialysis [4]. Additionally, elderly ESRD patients may present limitations in decision making because of dementia and other cognitive or physical conditions. Therefore, our study was performed across a wide spectrum of clinical outcomes that included survival rates, cardiovascular events, QOL, physical and mental performance, nutritional status, biochemical parameters, and residual renal function.

Our study has several limitations. First, this study did not consider the economic and ethical concerns regarding dialysis initiation. As with other life-sustaining therapies, RRT initiation in the elderly population also includes economic and ethical issues [32]. Compared with nondialytic conservative management, elderly ESRD patients who undergo dialysis are more likely to spend more of their remaining life years in dialysis centers or hospitals, and the likelihood of dying in a hospital is 2- to 3-fold higher than in hospice units or at home with family care [33]. If we could determine the period of time that lapses between an eGFR of 13.5 and 6.5 mL/min/1.73m^2^, which were the mean eGFRs of the early dialysis group and the late dialysis group, respectively, we could infer the corresponding economic and QOL benefits. To calculate this period, individual serial eGFR data would need to be available from pre-dialysis to dialysis initiation. Unfortunately, our cohort began enrolling patients from the start of dialysis. The Korean cohort study for outcomes in patients with chronic kidney disease (KNOW-CKD) enrolled approximately 2,450 adults with chronic kidney disease over a 5-year period from 2011 to 2015 [34]. The participating individuals will be monitored for approximately 10 years until death or ESRD. Analyses of the KNOW-CKD data would enable estimation of the benefit of late dialysis initiation. However, these data are not yet available. We focused on medical conditions because of the lack of medical evidence regarding the timing of dialysis initiation and the clinical outcomes in elderly ESRD patients. Second, our study included only Korean patients; therefore, generalizing to all patients preparing to undergo dialysis worldwide is difficult. Third, as our data were not generated from a RCT, subjects potentially had worse clinical conditions may have been included in the early dialysis group and those in better conditions could have been included in the late dialysis group, even after adjusting for multiple confounders and selection effects. Fourth, our cohort did not include data on the primary reasons for dialysis initiation. However, we conducted multivariate analyses and PSM to overcome the limitations of our cohort study. Additionally, we collected a variety of clinical parameters such as comorbidities and physical (e.g., vital signs, performance status), nutritional, biochemical (e.g., serum hemoglobin, albumin, potassium levels and BNP) and radiologic data, which could indicate the patient’s condition at the time of dialysis initiation. Based on these data, the early initiation group did not seem to be clinically worse than the late initiation group at the time of initiating dialysis. Fifth, the overall 1-year mortality was relatively high compared to rates in other developed countries and even to Korean national registry data. According to data from the Korean End-Stage Renal Disease Registry in 2015, the overall 1-year mortality of hemodialysis patients was 7.6%, and that of peritoneal dialysis patients was 6.9% [35]. However, our mortality data were from elderly patients. Therefore, compared with patients of all age groups, the mortality of our patients would be expected to be higher. In our subgroup analysis of subjects over 70 years in age (S2 Table), early dialysis showed significantly higher mortality in Model 1 and 4 (HRs: 1.82, 95% CI 1.24–2.65 and 1.91, 95% CI 1.16–3.16), and the HRs of these patients were higher than those of the whole study population (HRs: 1.70, 95% CI 1.26–2.89 and 1.46, 95% CI 0.98–2.20). These data suggest that early dialysis initiation poses a potential risk on patient survival in older population.

In conclusion, dialysis initiation timing is not associated with long-term survival in elderly patients with ESRD, and cardiovascular event-free survival, QOL, physical and mental performance, nutritional status, biochemical parameters, and residual renal function are also unaffected by the timing of dialysis initiation. We suggest delaying dialysis among elderly patients with ESRD as long as the patient's physical and mental abilities are not impaired and significant biochemical derangement has not occurred.

## Supporting information

S1 Fig Distribution of propensity scores of the patients before and after propensity score matching. The propensity scores of unmatched patients were significantly different between the early and late dialysis initiation groups, whereas the propensity scores of matched patients were nearly equivalent between the two groups.
(TIF)

S2 Fig Cumulative patient survival (A and B) and cardiovascular event-free survival (C and D) obtained using the Kaplan-Meier method in the older (over 70 years) subgroup of the cohort differentiated by the median estimated glomerular filtration rate before (A and C) and after (B and D) propensity score matching. (TIF)

S1 Table Dialysis information 3 momths after initiating dialysis. (DOCX)

S2 Table Multivariate risk factor analysis of the mortality of the older (over 70 years) subgroup of the cohort in the early dialysis group compared to the late dialysis group using Cox regression models and a propensity score matched model. (DOCX)
