# Supplementary figures and images for: Early dialysis initiation does not improve clinical outcomes in elderly end-stage renal disease patients: A multicenter prospective cohort study
Source: PLoS One. 2017 Apr 17;12(4):e0175830. doi: 10.1371/journal.pone.0175830 (PMC5393880; doi:10.1371/journal.pone.0175830)

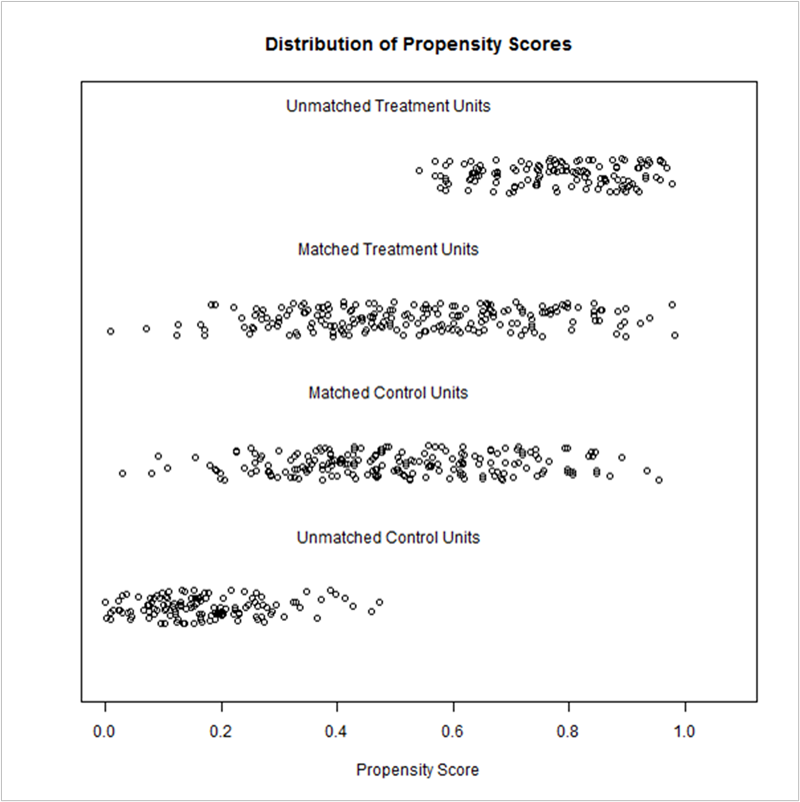

Supplement: S1 Fig — The propensity scores of unmatched patients were significantly different between the early and late dialysis initiation groups, whereas the propensity scores of matched patients were nearly equivalent between the two groups. (TIF) [file pone.0175830.s001.tif]

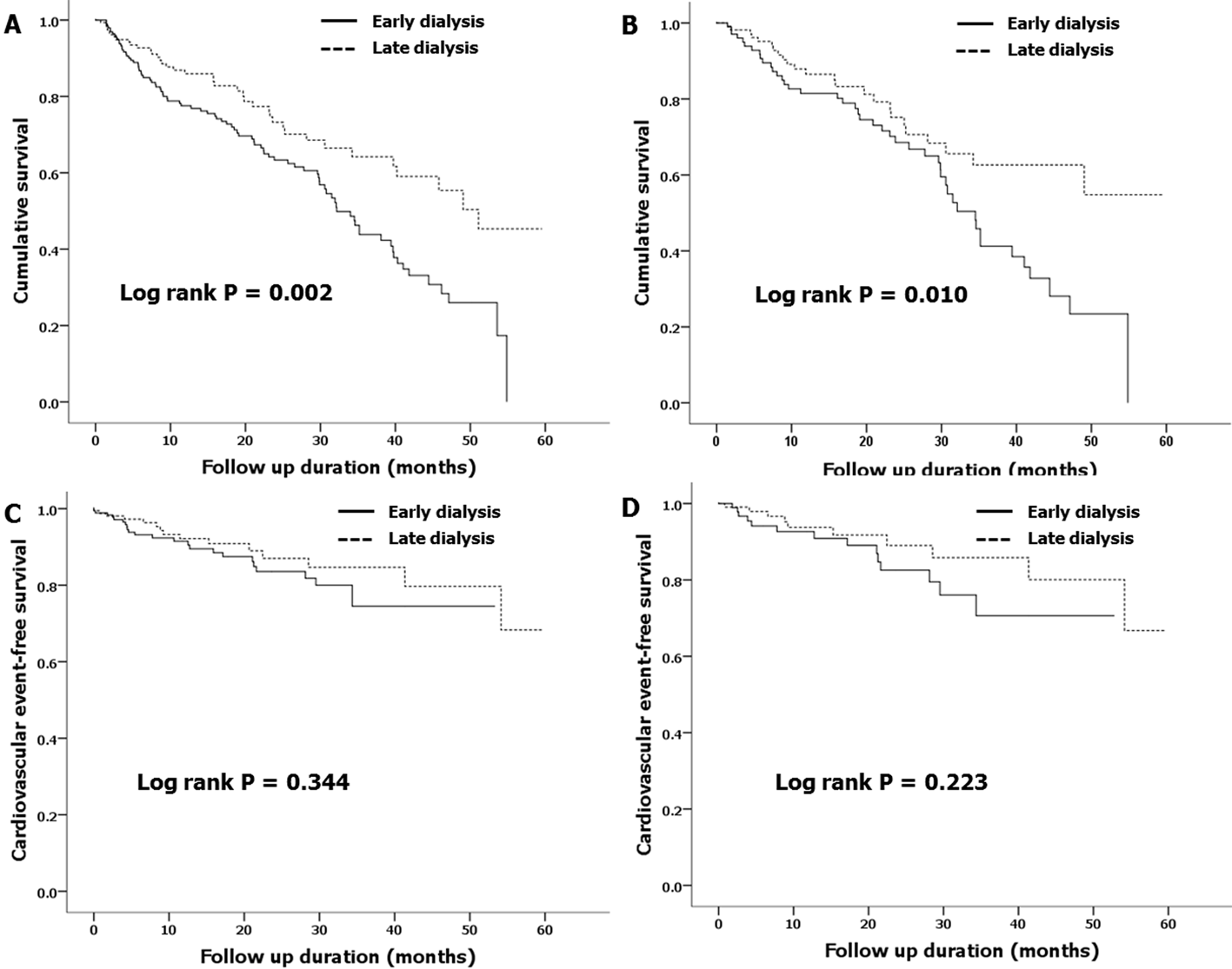

Supplement: S2 Fig — (TIF) [file pone.0175830.s002.tif]
