# Supplementary material for: Early dialysis initiation does not improve clinical outcomes in elderly end-stage renal disease patients: A multicenter prospective cohort study
Source: PLoS One. 2017 Apr 17;12(4):e0175830. doi: 10.1371/journal.pone.0175830 (PMC5393880; doi:10.1371/journal.pone.0175830)
Supplement: S1 Table — (DOCX) [file pone.0175830.s003.docx]

**S1 Table**. Dialysis information 3 months after initiating dialysis.

| Variables | Early dialysis (N=336) | Late dialysis (N=329) | *P* value |
| --- | --- | --- | --- |
| Dialysis frequency per week | 2.8 ± 0.5 | 2.9 ± 0.5 | 0.320 |
| Time per session (hours) | 3.9 ± 0.6 | 3.9 ± 0.5 | 0.888 |
| Urea reduction ratio (%) | 68.8 ± 8.7 | 69.6 ± 8.8 | 0.500 |
| Kt/V | 1.4 ± 0.3 | 1.5 ± 0.6 | 0.528 |
| Weekly Kt/V (only for PD patients) | 3.2 ± 2.7 | 3.1 ± 3.0 | 0.890 |
