# Supplementary material for: Early dialysis initiation does not improve clinical outcomes in elderly end-stage renal disease patients: A multicenter prospective cohort study
Source: PLoS One. 2017 Apr 17;12(4):e0175830. doi: 10.1371/journal.pone.0175830 (PMC5393880; doi:10.1371/journal.pone.0175830)
Supplement: S2 Table — (DOCX) [file pone.0175830.s004.docx]

**S2 Table.** Multivariate risk factor analysis of the mortality of the older (over 70 years) subgroup of the cohort in the early dialysis group compared to the late dialysis group using Cox regression models and a propensity score matched model.

|  | Hazard ratio | 95% confidence interval | *P* value |
| --- | --- | --- | --- |
| Model 1^a^ | 1.82 | 1.24-2.65 | 0.002 |
| Model 2^b^ | 1.48 | 1.00-2.20 | 0.051 |
| Model 3^c^ | 1.49 | 1.00-2.22 | 0.055 |
| Model 4^d^ | 1.91 | 1.16-3.16 | 0.011 |

^a^Unadjusted

^b^Adjusted for age, sex, and Charlson comorbidity index

^c^Adjusted for age, sex, Charlson comorbidity index, hemoglobin, albumin, calcium, and phosphorus

^d^Propensity score matched; covariates for matching: age, sex, Charlson comorbidity index, hemoglobin, albumin, calcium, and phosphorus
